# COVID-19 and hypertension: risks and management. A scientific statement on behalf of the British and Irish Hypertension Society

**DOI:** 10.1038/s41371-020-00451-x

**Published:** 2021-01-22

**Authors:** Christopher E. Clark, Sinead T. J. McDonagh, Richard J. McManus, Una Martin

**Affiliations:** 1 Primary Care Research Group, Institute of Health Services Research, College of Medicine & Health, University of Exeter Medical School, Smeall Building, St Luke’s Campus, Magdalen Road, Exeter, Devon, England EX1 2LU UK; 2 Nuffield Department of Primary Care Health Sciences, Radcliffe Primary Care Building, Radcliffe Observatory Quarter, University of Oxford, Woodstock Road, Oxford, England OX2 6GG UK; 3 Birmingham Medical School, Institute of Clinical Sciences, College of Medical and Dental Sciences, University of Birmingham, Birmingham, B15 2TT UK

**Keywords:** Risk factors, Hypertension

Hypertension is the single largest global contributor to disability-adjusted life years lost [1]. The majority of the population aged over 60 years have hypertension [2], and it has been suggested that they may be at increased risk from the effects of COVID-19. Despite this, and perhaps due to its ubiquity in the older population, current UK Government guidance does not identify people with hypertension as ʻat risk’ [3], however, other bodies such as the British Heart Foundation and the Health Service Executive in Ireland do [4, 5]. This article seeks to summarise and interpret the current evidence for and against an increase in COVID-19 risk and severity for those with raised blood pressure, and discusses the implications for the choice of anti-hypertensive treatment.

Early small case series did not indicate an excess of hypertension in people admitted to hospital with COVID-19 [6]. The Novel Coronavirus Pneumonia Emergency Response Epidemiology Team published a large case series from China; they found an overall case fatality rate of 2.3% (1023 of 44,672 confirmed cases), which increased to 6.0% for people with hypertension [7]. These data were reported without adjustment for age. Both COVID-19 case fatality rates and hypertension prevalence increase with age, reaching 8.0% and over 50% respectively for the 70–79 year age group [2]. New emerging evidence from the largest epidemiological study to date, examining over 17 million health records in England suggests that hypertension or a recorded blood pressure ≥140/90 mmHg taken together are not associated with COVID-19 in-hospital mortality after full adjustment: Hazard Ratio (HR) 0.95, (95% confidence interval (CI) 0.89–1.01). In sensitivity analyses, diagnosed hypertension alone was associated with slightly increased risk (HR 1.07, 95% CI 1.00–1.15) which might reflect residual confounding due to the strong age-related association [8].

Several other small case series that examine hypertension prevalence with and without severe COVID-19 have appeared in the literature [9]. In March 2020, a study-level meta-analysis of 2552 confirmed COVID-19 patients reported a pooled odds ratio (OR) of 2.49 (CI: 1.98–3.12; 11 studies) for severe disease in the presence of hypertension, with low heterogeneity between studies (*I*^2^ = 24%). The OR for death was similar and weak evidence from meta-regression suggested that hypertension might be a clinical predictor of COVID-19 severity in people aged over 60 [10]. Likewise, a retrospective cohort analysis of 191 patients treated in hospital in China (not included in the meta-analysis) confirmed apparent high mortality in patients presenting with hypertension: 48% versus 23% of survivors [11].

Similar findings were reported with previous coronavirus infections, such as severe acute respiratory syndrome and Middle East Respiratory Syndrome [12, 13]. The mechanism by which hypertension leads to increased risk from COVID-19 is undoubtedly complex and may well relate to underlying co-morbidity. The prognosis for people with hypertension is markedly worse when COVID-19 infection is complicated by myocardial injury and in the presence of cardiovascular disease [8, 14]. End organ damage and cardiovascular events are associated with poorer control of high blood pressure, and mean blood pressure rises with age [15, 16]. It seems plausible, therefore, that older age, poorer blood pressure control and cardiovascular disease can explain the observed associations between age, hypertension and severity of COVID-19 infection.

## Are anti-hypertensive medications a risk for severity of COVID-19?

The viral structural spike (S) protein binds to the angiotensin-converting enzyme 2 (ACE2) receptor to gain entry to cells; this has led to suggestions that use of ACE inhibitors and angiotensin II type-I receptor blockers (ARBs) may be implicated in poorer outcomes with COVID-19 [17, 18]. Whilst gaining much publicity, with attendant worry for patients and clinicians, this theory has been debated and ACE inhibitor and ARB treatment has also been associated with improved outcomes in some reports [19, 20]. Polymorphism in expression of the ACE2 receptor gene in association with hypertension and excess cardiovascular risk in ethnic minority groups has also been suggested as a partial explanation for observed excess mortality from COVID-19 in these populations [21, 22]. The National Institute for Health and Care Excellence are currently undertaking a rapid review of this topic [23]. Recent observational studies of confirmed COVID-19 patients have increased confidence that these drugs are not harmful: a case-control study of 6272 COVID-19 patients in Lombardy, Italy, matched to 30,759 controls, confirmed higher use of ACE inhibitors and ARBs in patients than controls, but after adjustment for greater prevalence of underlying cardiovascular disease, there was no evidence that ACE inhibitors (OR 0.96; 95% CI 0.87–1.07) or ARBs (OR 0.95; 95% CI 0.86–1.05) were associated with different risks of COVID-19 [24]. Two large retrospective cohort studies have also been published; one found no association between ACE inhibitor or ARB use and a positive coronavirus test in 18,472 people from Ohio and Florida (weighted OR 0.97; 95% CI 0.81–1.15), whist another study of 12,594 electronic health records from New York, found no association between *any* anti-hypertensive medication (ACE inhibitors, ARBs, beta-blockers, calcium-channel blockers, or thiazide diuretics) and increased likelihood of either a positive coronavirus test or an increased risk of severe COVID-19 illness [25, 26].

It is important to recognise that, to date, no randomised trial evidence exists to demonstrate either benefits or risks of continuing ACE inhibitors or ARBs on the incidence or outcomes of COVID-19. It must also be acknowledged that these observational data carry risks of residual confounding [27]. One retrospective cohort study found a positive association between ACE inhibitor or ARB use and admission to hospital (OR 1.93; 95% CI 1.38–2.71) or intensive care (OR 1.64; 95% CI 1.07–2.51) in small subgroup analyses (*N* = 421 and 161, respectively) [26]. These secondary outcomes are rightly viewed with caution due to the potential for residual confounding by co-morbidity and also imprecision (as evidenced by wide confidence intervals) due to small sample sizes [28]. Nevertheless, these studies taken together convey a consistent message of absence of harm associated with anti-hypertensive medications during infection with coronavirus, and clinical equipoise should therefore apply. Whilst these drugs appear increasingly unlikely to worsen COVID-19 severity, the consequences of poor blood pressure control, under normal circumstances, are well documented [16]. Consequently, the British and Irish Hypertension Society (BIHS), and our European and International partner societies, have issued clear position statements advising against cessation of anti-hypertensive therapy on the grounds of concern over risks with COVID-19 (available at: https://bihsoc.org/bihs-statement-on-acei-arb-and-covid19/) [29]. Current evidence does not support published opinions that doctors may consider stopping treatment with ACE inhibitors and ARBs in well-controlled patients with mild (Stage 1) hypertension and coronavirus infection [30]. The unintended consequences of discontinuing effective treatments for hypertension, without a suitable replacement titrated against appropriate blood pressure measurements and under direct medical supervision, could put patients at increased cardiovascular risk. In addition, managing such titration at a time when primary care is prioritising acute illness over routine contacts (including surgery blood pressure checks), makes the proposed strategy impractical and risks further diluting access to care.

## So, what can we say to hypertensive patients who may be anxious about taking anti-hypertensive medication and about their risks from infection during the COVID-19 pandemic?

The evidence base remains incomplete, so strong recommendations are difficult. However, people with complications of hypertension, such as ischaemic heart disease, are already regarded as being at high risk from COVID-19. It seems reasonable to advise those with poorly controlled hypertension (i.e. blood pressure above guideline targets), particularly if prolonged, to also consider themselves to be at elevated risk, and, therefore, to follow appropriate social distancing advice [3]. For younger individuals with hypertension, there is an association of obesity with COVID-19 severity among Western populations [2, 8, 31, 32]. Generally, for people with good control of blood pressure, risks of undiagnosed cardiovascular disease are low, and they could therefore be reassured. All hypertensive patients should be strongly reassured that continuing their current medications is both safe and desirable.

We should support all our hypertensive patients in continuing to strive for, and maintain, good blood pressure control by continuing to take their medications as prescribed, and by endeavouring to follow and maintain sensible lifestyle choices, including regular exercise [3].

Large numbers of people are being affected by COVID-19 with potentially profound consequences, however, their continued surveillance will also provide further data that will help us to understand the true risk from elevated blood pressure, its treatment and co-morbidities, on outcomes of the disease. Careful and continuous research is vital for an understanding of the mechanisms underlying any additional risk from hypertension with COVID-19, and to determine the best and safest ways to treat those with severe manifestations of disease. Our patients are best served when their treatment is based on available evidence rather than speculation, and the growing body of evidence suggests that continuation of ACE inhibitors and ARBs during the current COVID-19 pandemic is safe.

## References

[1] Global Burden of Disease 2015 Risk Factors Collaborators. Global, regional, and national comparative risk assessment of 79 behavioural, environmental and occupational, and metabolic risks or clusters of risks, 1990–2015: a systematic analysis for the Global Burden of Disease Study 2015. Lancet. 2016;388:1659–724. 10.1016/S0140-6736(16)31679-8 PMC5388856 27733284

[2] Kearney PM, Whelton M, Reynolds K, Muntner P, Whelton PK, He J (2005). Global burden of hypertension: analysis of worldwide data. Lancet.

[3] Who’s at higher risk from coronavirus. https://www.nhs.uk/conditions/coronavirus-covid-19/people-at-higher-risk/whos-at-higher-risk-from-coronavirus/ NHS England 7/1/21; accessed 18 Jan 2021.

[4] Health Service Executive. At-risk groups: coronavirus (COVID-19). https://www2.hse.ie/conditions/coronavirus/at-risk-groups.html; 5/4/20; accessed 6 Apr 2020.

[5] British Heart Foundation. Coronavirus: what it means for you if you have heart or circulatory disease. https://www.bhf.org.uk/informationsupport/heart-matters-magazine/news/coronavirus-and-your-health 3/4/20; accessed 6 Apr 2020.

[6] Huang C, Wang Y, Li X, Ren L, Zhao J, Hu Y (2020). Clinical features of patients infected with 2019 novel coronavirus in Wuhan, China. Lancet.

[7] The Novel Coronavirus Pneumonia Emergency Response Epidemiology T (2020). The epidemiological characteristics of an outbreak of 2019 novel coronavirus diseases (COVID-19) — China, 2020. China CDC Weekly.

[8] Williamson E, Walker AJ, Bhaskaran KJ, Bacon S, Bates C, Morton CE, et al. OpenSAFELY: factors associated with COVID-19-related hospital death in the linked electronic health records of 17 million adult NHS patients. medRxiv. 2020. 10.1101/2020.05.06.20092999.

[9] Chen T, Wu D, Chen H, Yan W, Yang D, Chen G (2020). Clinical characteristics of 113 deceased patients with coronavirus disease 2019: retrospective study. BMJ.

[10] Lippi G, Wong J, Henry BM (2020). Hypertension in patients with coronavirus disease 2019 (COVID-19): a pooled analysis. Pol Arch Intern Med.

[11] Zhou F, Yu T, Du R, Fan G, Liu Y, Liu Z (2020). Clinical course and risk factors for mortality of adult inpatients with COVID-19 in Wuhan, China: a retrospective cohort study. Lancet.

[12] Badawi A, Ryoo SG (2016). Prevalence of comorbidities in the Middle East respiratory syndrome coronavirus (MERS-CoV): a systematic review and meta-analysis. Int J Infect Dis.

[13] Wong WW, Chen TL, Yang SP, Wang FD, Cheng NC, Kuo BI (2003). Clinical characteristics of fatal patients with severe acute respiratory syndrome in a medical center in Taipei. J Chin Med Assoc.

[14] Guo T, Fan Y, Chen M, Wu X, Zhang L, He T (2020). Cardiovascular implications of fatal outcomes of patients with coronavirus disease 2019 (COVID-19). JAMA Cardiol.

[15] The SPRINT Research Group. A randomized trial of intensive versus standard blood-pressure control. N Engl J Med. 2015;373:2103–16. 10.1056/NEJMoa1511939 PMC4689591 26551272

[16] Lewington S, Clarke R, Qizilbash N, Peto R, Collins R (2002). Age-specific relevance of usual blood pressure to vascular mortality: a meta-analysis of individual data for one million adults in 61 prospective studies. Lancet.

[17] Sanders JM, Monogue ML, Jodlowski TZ, Cutrell JB (2020). Pharmacologic treatments for coronavirus disease 2019 (COVID-19): a review. JAMA.

[18] Fang L, Karakiulakis G, Roth M (2020). Are patients with hypertension and diabetes mellitus at increased risk for COVID-19 infection?. Lancet Respir Med.

[19] Meng J, Xiao G, Zhang J, He X, Ou M, Bi J (2020). Renin-angiotensin system inhibitors improve the clinical outcomes of COVID-19 patients with hypertension. Emerg Microbes Infect.

[20] Vaduganathan M, Vardeny O, Michel T, McMurray JJV, Pfeffer MA, Solomon SD (2020). Renin-angiotensin-aldosterone system inhibitors in patients with covid-19. N Engl J Med.

[21] Khunti K, Singh AK, Pareek M, Hanif W (2020). Is ethnicity linked to incidence or outcomes of covid-19?. BMJ.

[22] Lu R, Zhao X, Li J, Niu P, Yang B, Wu H (2020). Genomic characterisation and epidemiology of 2019 novel coronavirus: implications for virus origins and receptor binding. Lancet.

[23] National Institute for Health and Care Excellence. Coronavirus (COVID-19). 2020. https://www.nice.org.uk/covid-19; accessed 6 Apr 2020.

[24] Mancia G, Rea F, Ludergnani M, Apolone G, Corrao G. Renin–angiotensin–aldosterone system blockers and the risk of covid-19. N Engl J Med. 2020;382:2431–40. 10.1056/NEJMoa2006923 PMC7206933 32356627

[25] Reynolds HR, Adhikari S, Pulgarin C, Troxel AB, Iturrate E, Johnson SB, et al. Renin–angiotensin–aldosterone system inhibitors and risk of covid-19. N Engl J Med. 2020;382:2441–8. 10.1056/NEJMoa2008975 PMC7206932 32356628

[26] Mehta N, Kalra A, Nowacki AS, Anjewierden S, Han Z, Bhat P. Association of use of angiotensin-converting enzyme inhibitors and angiotensin II receptor blockers with testing positive for coronavirus disease 2019 (COVID-19). JAMA Cardiol. 2020;5:1020–6. 10.1001/jamacardio.2020.1855 PMC7201375 32936273

[27] Jarcho JA, Ingelfinger JR, Hamel MB, D’Agostino RB, Harrington DP (2020). Inhibitors of the renin–angiotensin–aldosterone system and covid-19. N Engl J Med.

[28] Thomas LE, Bonow RO, Pencina MJ (2020). Understanding observational treatment comparisons in the setting of coronavirus disease 2019 (COVID-19). JAMA Cardiol.

[29] British and Irish Hypertension Society. BIHS statement on ACEi/ARB and COVID-19. 2020 https://bihsoc.org/bihs-statement-on-acei-arb-and-covid19/; accessed 6 Apr 2020.

[30] Aronson JK, Ferner RE (2020). Drugs and the renin-angiotensin system in covid-19. BMJ.

[31] Kass DA, Duggal P, Cingolani O. Obesity could shift severe COVID-19 disease to younger ages. The Lancet 4/5/20. 10.1016/S0140-6736(20)31024-2 (accessed 12 May 2020.). 10.1016/S0140-6736(20)31024-2 PMC7196905 32380044

[32] Sattar N, McInnes IB, McMurray JJV. Obesity a risk factor for severe covid-19 infection: multiple potential mechanisms. Circulation. 2020 22/4/20; https://www.ahajournals.org/doi/abs/10.1161/CIRCULATIONAHA.120.047659 (accessed 12 May 2020.). 10.1161/CIRCULATIONAHA.120.047659 32320270

